# Global photovoltaic solar panel dataset from 2019 to 2022

**DOI:** 10.1038/s41597-025-04985-y

**Published:** 2025-04-16

**Authors:** Anqi Li, Luling Liu, Shijie Li, Xihong Cui, Xuehong Chen, Xin Cao

**Affiliations:** 1 State Key Laboratory of Remote Sensing and Digital Earth, Faculty of Geographical Science, Beijing Normal University, Beijing, 100875 China; 2 Beijing Engineering Research Centre for Global Land Remote Sensing Products, Faculty of Geographical Science, Beijing Normal University, Beijing, 100875 China; 3 Moganshan Geospatial Information Laboratory, Deqing, 313200 China

**Keywords:** Energy management, Energy supply and demand, Sustainability

## Abstract

Solar photovoltaic (PV) power generation, known for its affordability and environmental benefits, is a key component of the global energy supply. However, the lack of comprehensive, timely, and precise global PV datasets has limited spatial analysis of PV potential. We developed a new method to identify PV panels globally, producing an annual 20-meter resolution dataset for 2019–2022. This dataset offers unprecedented detail and accuracy for future research and policy-making. A two-stage PV classification framework was built using U-Net and positive unlabelled learning with random forest (PUL-RF). U-Net first recognizes PVs from sub-meter Google Earth images, expanding positive PV samples for the second stage, where PUL-RF classifies Sentinel-2 images on a large scale. The dataset was evaluated with IoU and F1-Score metrics, achieving over 90% accuracy. Compared to existing datasets, it provides better precision and spatial detail, showing global PV growth of over 60% between 2019 and 2022, with developing countries leading the increase.

## Background & Summary

In order to realize a long-lasting, safe, and reliable supply of energy, countries around the world are stepping up the development of renewable energy power generation technologies to continuously promote the green and low-carbon energy transition^1,2^. Solar photovoltaic (PV) power generation systems not only have access to the most abundant resources but also possess the cleanest power generation process, which can be used as an alternative to fossil fuels to address the energy supply gap in the context of the global energy crisis^3–5^, and therefore became a renewable energy generation technology with the most desirable characteristics for sustainable development^6–8^. According to the data provided by the International Energy Agency (IEA), the global new installed capacity of photovoltaics in 2022 was 133 GW, while the cumulative installed capacity reached 843.0 GW. In 2023, global installed renewable energy capacity additions are expected to increase by 50% compared to 2022. This marks the fastest growth in installed capacity in the past 30 years, with a period of rapid expansion predicted for the next five years^9^. It has been pointed out that with the development of PV power output technology and the decreasing cost of PV power generation, the number of PV power plants is growing at a rapid pace, and there is a huge demand for PV power in the electricity market^10^. These reports and studies point out that PV is maintaining a growing trend and has a very bright future. As a result, the huge installed PV potential has led to an increasing focus on PV scale, newly installed capacity, and PV power output; therefore, accurate geospatial and power information, such as location, size, capacity, and power output, are particularly important in the PV development process.

Research on PV power generation is currently growing rapidly, with studies extracting PV solar panel locations and PV array sizes emerging^11,12^. However, the available PV mapping datasets are with limited spatial range or lack of timely updating (Table 1), which greatly hinders researchers from exploring global PV solar panel size, developmental changes, and power generation. As a result, existing studies often lack spatial data support when analyzing the power supply potential of PV. Most of the studies only summarize the development trend of PV power generation in terms of socio-economic and national policies^13–15^ and lack a geospatial perspective to carry out research on spatial and temporal variations in the development of PV.

**Table 1:** The available PV distribution datasets and shortcomings.

PV data set	Description	Limitations
Global Solar 2020^16^	Global PV point vector dataset (2020)	Only the PV farm locations was recorded, without any shape information
DeepSolar database^28^	The US PV dataset with a resolution of 0.15 meters before 2018	Limited spatial range
China PV plants^12^	The Chinese PV dataset with a resolution of 30 meters in 2020	Limited spatial range
SolarNet database^29^	439 PV power plants within China in 2019	Limited spatial range
Global solar units^30^	Global PV dataset from 2016/06/01 to 2018/09/30	No updates in recent years

It can be seen that global PV extraction and further acquisition of global PV distribution datasets are beneficial for grasping global PV dynamics from a spatial perspective. For global PV solar panel monitoring and tracking, the most important information is the location information of PV power stations. The latest publicly released global PV plant location data in latitude and longitude is Global_Wind_Solar_2020^16^, which was developed with OpenStreetMap as a support^17^ and contains the 2020 global location of wind turbines and PV farms information in latitude and longitude coordinates. The same dataset is also available as the Wiki-Solar dataset^18^. These data lack a spatially-based extraction method to visualize the scale information of PV solar panels and thus have some limitations in their use. With the development of remote sensing technology, more and more studies use remote sensing monitoring for PV solar panel extraction^19–21^, which can be categorized into categories such as machine learning and deep learning based on the methods. Some scholars put the spectral information of PV solar panels as features into a random forest classifier (RF) for training as a way to realize solar panel extraction^12^, and a 30-meter resolution dataset of PV power plants in China in 2020 was produced using a random forest classifier based on Landsat 8 imagery. RF has the following characteristics advantages of high accuracy, efficiency, and stability^22–24^, which enables the extraction of PV solar panels. However, RF has a high dependence on samples, so RF has not been applied to extract PV globally in previous studies. In recent years, the rapid development of deep learning has made researchers more and more inclined to adopt deep learning models for feature classification^25–27^, whose classification methods can also be used for PV solar panel extraction. The use of a Convolutional Neural Network (CNN) enabled the design of DeepSolar^28^, a deep learning model for PV solar panel detection, to obtain the distribution of PV solar panels within the United States. Some researchers have also used U-Net to design the deep learning framework SolarNet^29^, which extracted 439 PV power plants within China based on Google Earth images. Some scholars also used deep learning models such as U-Net, ResNet, and RNN for global PV solar panel mapping in 2018 based on SPOT and Sentinel-2 images, and this study processed a total of 550 TB of images^30^. It can be seen that deep learning methods are equally effective in recognizing solar PV installations, but the huge data storage and arithmetic power in the deep learning process are constraints. The above problems have led to the fact that global datasets of PV solar panels with accurate mapping, high timeliness, and long-time series are still scarce.

The key problem we need to solve is to fully combine the advantages of machine learning and deep learning so that the classifiers can ensure the high accuracy of deep learning in semantic segmentation tasks. At the same time, machine learning can be used to save the arithmetic and time costs consumed by deep learning. To address these challenges, we propose a two-stage classification framework that integrates deep learning and machine learning classifiers. This approach is based on the concept of utilizing multi-source remote sensing data and multiple classifiers. It aims to overcome issues such as the difficulty of sample selection in PV extraction, the inefficiency of recognition methods, and the lack of timeliness in existing PV power station datasets. In the first stage, we use the U-Net to produce a large and accurate number of positive PV samples for sample expansion. In the second stage, positive unlabelled learning with random forest (PUL-RF) is used to expand the extraction scale of PV to achieve more efficient extraction of PV solar panels globally and to realize the sustainable updating of the PV dataset. Based on this methodology, the production of a global PV dataset for 2019–2022 is accomplished. In this way, we can produce accurate mapping, time-sensitive and long-time series datasets, estimate the installed PV capacity of each country in the world, and analyze the characteristics of the global PV temporal and spatial variations.

In total, we have designed a more efficient and suitable two-stage classification framework for identifying PV on a global scale and produced a global annual 20-meter resolution PV dataset from 2019 to 2022. This will provide effective data support for PV development studies from a geospatial perspective.

## Methods

**Fig. 1:**
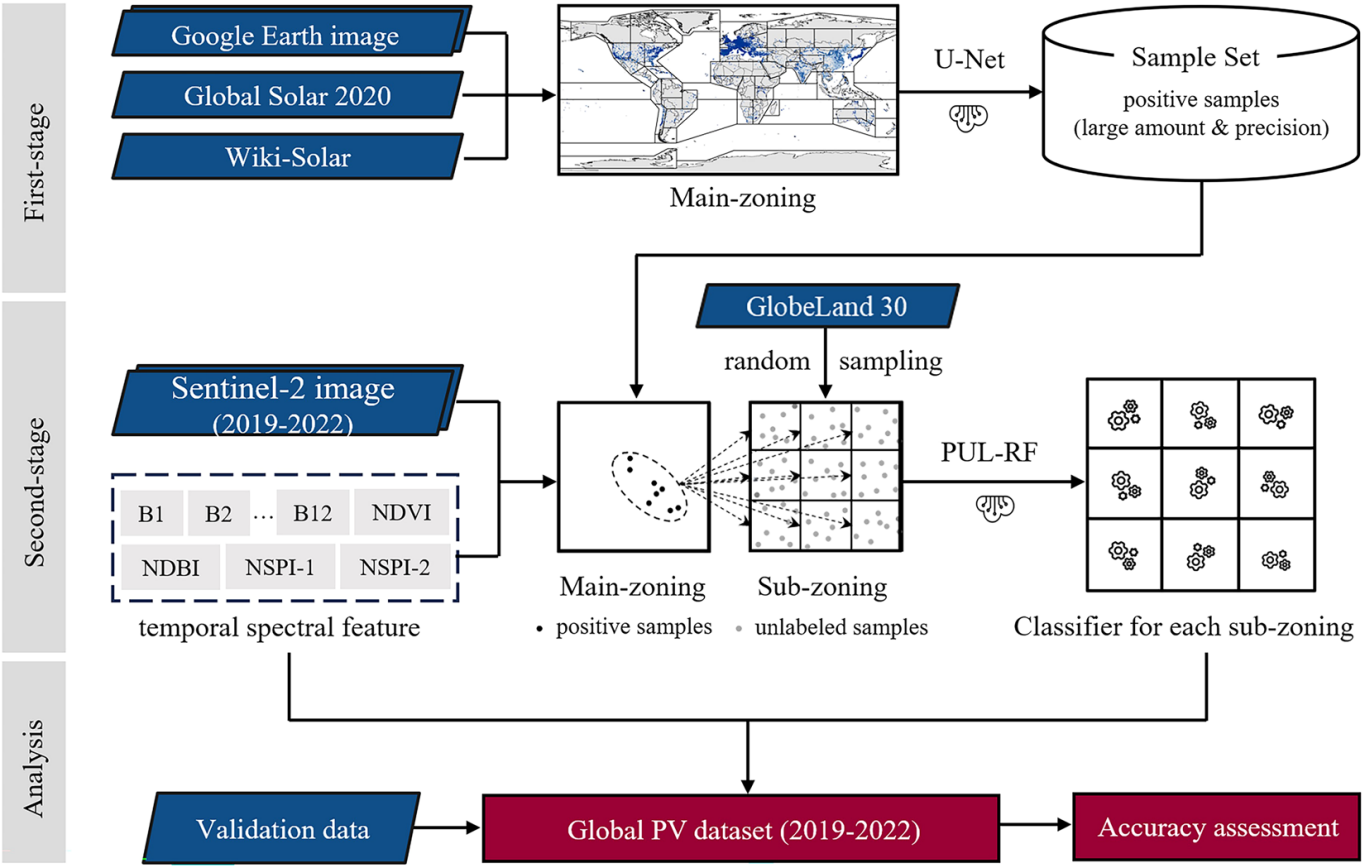
Flowchart of the two-stage classification framework.

We proposed a two-stage classification framework to extract PV solar panels globally (Fig. 1). In the first stage, a deep learning U-Net model is trained for extracting PVs from selected high-resolution (0.6 m) Google Earth (GE) images. The first stage classification aims to expand the pool of positive PV samples, which are used as the training samples for the second stage classification. In the second stage classification, we employed PUL-RF trained by the expanded PV sample set to classify Sentinel-2 images, enabling efficient classification at global scale. Moreover, a two-level zoning strategy is introduced in the process of realizing the two-stage classification framework, which helps to alleviate the intra-class variability of PV and non-PV features for different geographical conditions. Using the proposed two-stage classification framework, the annual global PV maps from 2019 to 2022 are produced and subsequently evaluated against a set of independent validation data.

### Data & data preprocessing

Multiple geographic information datasets (Table 2) are used in both the establishment and validation of the proposed two-stage classification framework for global PV mapping.

**Table 2:** A brief description of the data used in this study.

Data set	Role	Description
Global Solar 2020^16,31^	Provide the potential location of expanded PV samples	Point vector data of PV farm locations
Wiki-Solar dataset^32^	Provide the potential location of expanded PV samples	Point vector data of PV farm locations
High-resolution annotated PV samples^33^	Used for training U-Net in the first-stage classification	179 GE images (0.6 m) and manually annotated PV polygons.
Google Earth image^33^	Used in the first-stage classification process to generate PV samples for training PUL-RF in the second stage classification	1326 GE images (0.6 m) randomly selected nearby PV farms
Sentinel-2 data	Used in the second-stage classification	Multi-spectral remote sensing time-series with global coverage (20 m)
Land cover dataset^35,36^	Provide land cover types for stratified sampling the unlabelled samples	Global land cover data at 30 m resolution in 2020, with ten land cover types
Validation Data^38^	Accuracy evaluation	Including unchanged PV polygons and newly added PV polygons from 2019 to 2022

### Global PV farm locations dataset

The most widely used dataset for global PV farm locations is Global Solar 2020^16,31^, which we used to find the locations of PV power stations, but this dataset slightly misses the statistics of centralized PV. Therefore, we also introduced the Wiki-Solar dataset^18,32^ as a supplement. Global Solar 2020 contains 35272 vector points, and Wiki-Solar contains 1404 vector points. These point vectors represent the location information of the PV farms, lacking of the spatial presentation of the shape and size of the PV panels. Moreover, there is a slight deviation between the recorded location and the actual location. Therefore, the above two datasets cannot be directly used as samples, but can provide the potential locations of PV samples.

### High-resolution annotated PV sample set

We collected 122 GE images (0.6 m resolution, 1 km × 1 km area) and 57 GE images (0.6 m resolution, 2 km × 2 km area), with PVs and diverse land cover types, to establish our sample set^33^. To ensure the representativeness, we selected GE images with PVs installed on different land cover types including cultivated land, forest, artificial surfaces, deserts, mountains, and water bodies. Negative class samples that are close to the texture of PV solar panels and easy to confuse, such as parking lots, crosswalks, and dense buildings, are also added. Then we manually annotated PV polygons in these images. This annotated dataset is used for training U-Net model in the first-stage classification.

### Images from Google Earth platform

Google Earth (GE) images are satellite images that can be accessed through the Google Earth platform. GE images are generated by mosaicking together a large number of high-resolution satellite and aerial images. Sub-meter scale GE images are able to clearly show the alignment and texture information of PV solar panels, which were thus suitable for identifying PVs by deep learning models^29^. In this study, therefore, we only select a small subset of GE images for the first stage classification, which serves to expand PV sample set for the second stage classification. We used GE images with a resolution of around 0.53–0.54 m, and in order to unify the resolution of the images during use, we resampled them to 0.6 m^34^. By referring the point vectors from Global Solar 2020 and Wiki-Solar, we randomly downloaded 1326 GE images^33^ (0.6m resolution, 1 km × 1 km area, closest available date to 1 January 2021) around each vector point. It is noticed that we downloaded at least 44 GE images in each main zone, ensuring the representativeness of all of the main zones.

### Sentinel-2 imagery

The Sentinel-2 satellite carries the Multispectral Imager (MSI), which allows for systematic global land photography and is used to capture high-resolution imagery of the land surface. As a result, Sentinel-2 imagery is often used for surface detection and feature classification, and Sentinel-2 has multispectral imagery with a 5-day re-entry period covering the visible, near-infrared, and short-wave infrared spectral ranges and providing different resolutions in different wavelength bands.

**Fig. 2:**
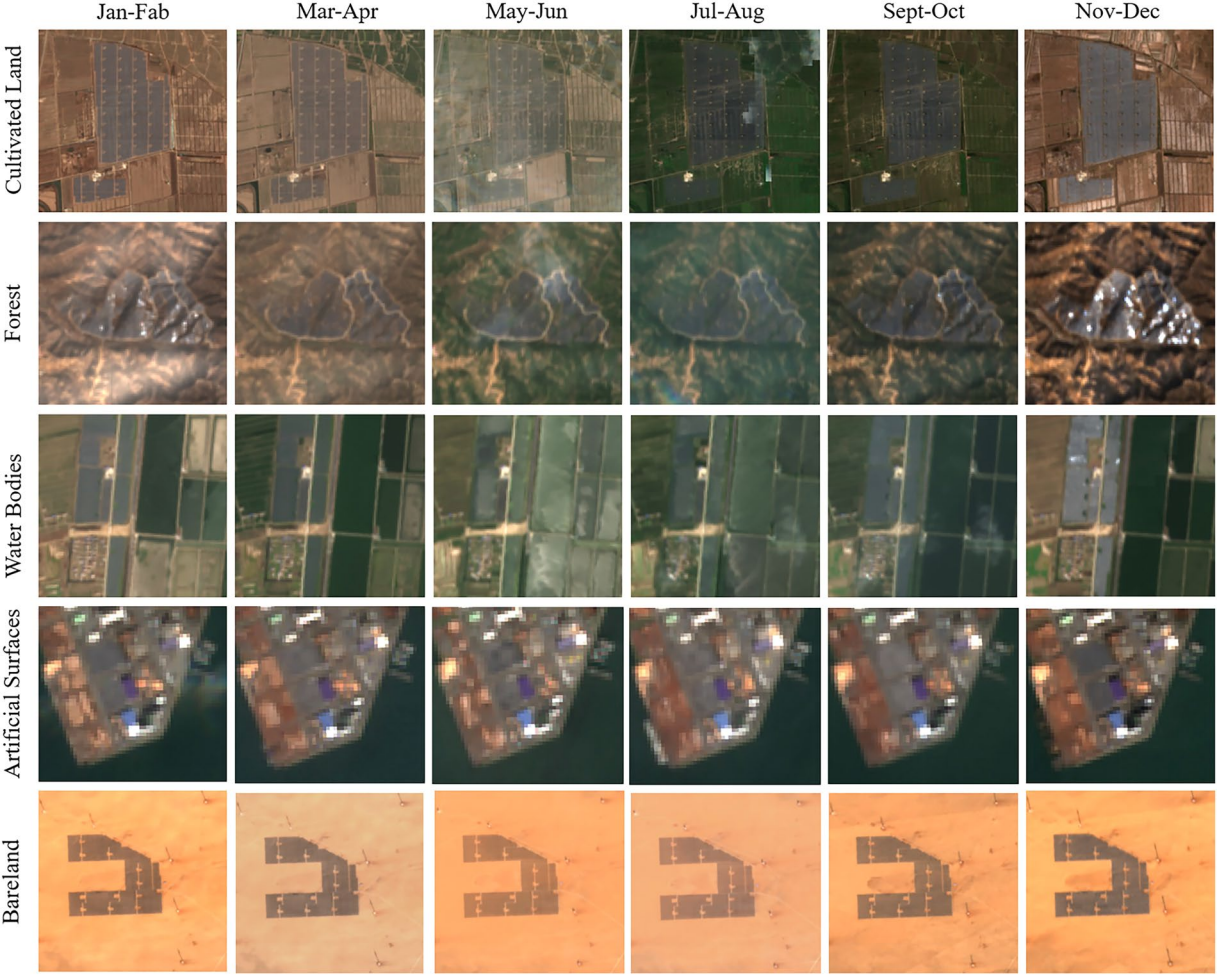
PV solar panels displayed in 2021 Sentinel-2 RGB image (median per bi-monthly composite).

After bi-monthly synthesis of the Sentinel-2 image in 2021, it can be observed that the PV solar panels do not change with the seasons, but other land surfaces, such as cultivated land and forested land, show changing characteristics (Fig. 2). The resolution of the visible band of the Sentinel-2 image is 10 m, the resolution of the near-infrared band is 20 m, and the resolution of the short-wave infrared is 60 m. This is very important data support for the identification of global PV solar panels, which have the advantages of multi-spectral band, high resolution, and full coverage. It can be seen that the Sentinel-2 image is characterized by multi-spectral bands, high resolution, strong timeliness, and full coverage, which provides important data support for global PV solar panel identification. We use the 2019–2022 Sentinel-2 images in the classification stage to extract the PV solar panels by their spectral information in the multispectral data.

We acquired and processed the Sentinel-2 L2A surface reflectance data based on the Google Earth Engine (GEE) cloud platform. The Sentinel-2 imagery on GEE was de-clouded by the QA band, and a bi-monthly median was used to synthesize time-series data with two-month intervals.

### Land cover dataset

The land cover data we use is the GlobeLand30 dataset from 2020 (GlobeLand30-2020), which provide rich and detailed global surface cover information at a resolution of 30 m in the base year of 2020, covering ten major surface cover types^35,36^. We use the GlobeLand30-2020 data for the collection of unlabelled class samples needed in the classification process, which will result in a richer type of unlabelled class samples to provide sample support for the one-class classifier.

In addition, we use the world map provided by the Resource and Environmental Science Data Platform^37^ as the administrative base map.

### Validation data

The validation dataset comprises two parts, unchanged PV samples from 2019 to 2022 and the newly added PV samples since 2019^38^. The unchanged PV samples were refined from ground validation data provided by Kruitwagen *et al*.^30^, which includes PV polygons of varying sizes installed before 2018. To ensure compatibility with our study period and the spatial resolution of Sentinel-2 data, we excluded the PV polygons that disappeared after 2019 and those smaller than a 20 m pixel, through visual interpretation of Sentinel-2 images. This refinement process yielded 600 accurate unchanged PV polygons from 2019 to 2022, which are used to evaluate the classification accuracy of our produced solar PV maps. The other part of our validation data consists of the newly added PV polygons since 2019. Through careful visual interpretation on Sentinel-2 images, we manually selected 200 new PV polygons for each of following time intervals: 2019 to 2020, 2020 to 2021, and 2021 to 2022. Thus, a total of 600 newly added PV polygons were obtained during the study period to assess the changes detection accuracy of our annually solar maps.

### Two-level zoning

For global studies, there is geographic variability in different regions, which makes global categorization a difficult task, and the difficulty is mainly in the selection of samples and the training of classifiers. For this reason, we propose the idea of two-level zoning considering the variability of global natural conditions.

**Fig. 3:**
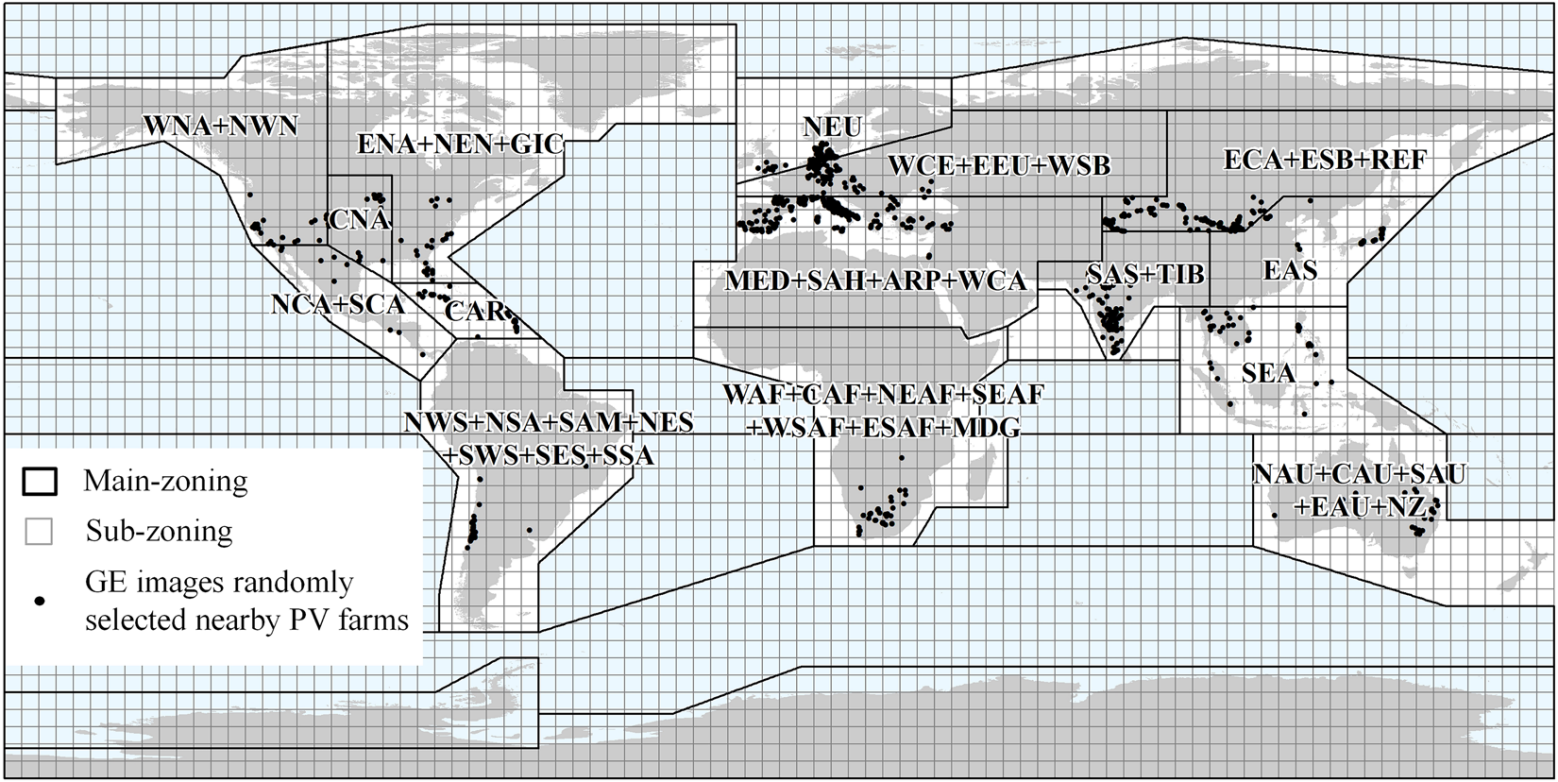
Schematic diagram of two-level zoning.

For the first level of zoning, the globe was zoned using the global climate zoning methodology used in IPCC AR6 WGI^39,40^, with each climate zoning as main-zoning, and PV positive samples were selected in main-zoning. Considering some main zones have very few PV samples, they are merged with other main zones (Fig. 3). Extracting PV solar panels globally is actually a binary classification problem that categorizes remote sensing images into PV and non-PV. For the binary classification problem, PV solar panels account for a very small percentage of the Earth’s surface, and in order to make the non-PV samples cover as many more comprehensive feature types as possible and to satisfy the balanced sample ratio of PV and non-PV, second-level zoning is needed to divide the area of the classified region into smaller areas as much as possible. The second level of zoning divides each main-zoning into a 4-degree × 4-degree grid, which forms a sub-zoning (Fig. 3). Positive samples from the main-zoning and unlabelled samples from the sub-zoning are used to classify each sub-zoning, and the classifier is trained in each sub-zoning.

### Two-stage classification framework

#### First-stage classification: PV samples generation based on U-Net

**Fig. 4:**
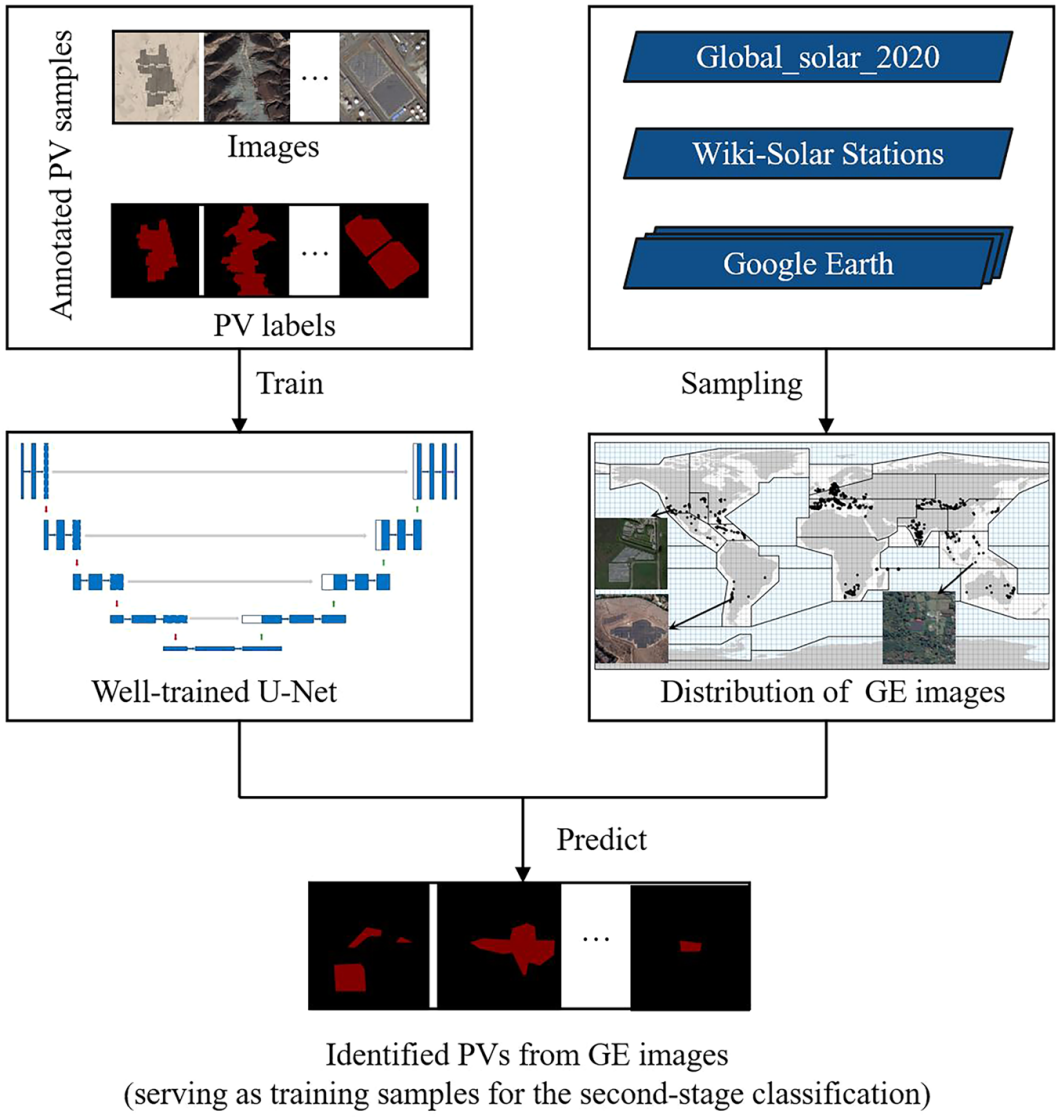
Flowchart of the first stage classification.

The first-stage classification is designed to generate adequate PV samples in each main zone (Fig. 4). Firstly, the high-resolution annotated PV sample set was used for training an U-Net for identifying PV polygons from GE images. The annotated sample set is divided into 70% training and 30% validation samples to establish the optimal U-Net model for PV extraction of GE images. U-Net is applied here considering its effectiveness in feature segmentation of remote sensing images in recent years^41–45^. Then, the well-trained U-Net is applied for extracting PV polygons from 1326 GE images across the world. These extracted PV polygons are then used for the training samples in the second stage classification.

#### Second-stage classification: global PV extraction based on PUL-RF

In the second-stage classification, we classify PV pixels from Sentinel-2 time-series through specifically trained PUL-RF (a typical one-class classifier) for each subzone (Fig. 1). We employ PUL-RF in this study, considering its efficiency and effectiveness in one-class mapping. PUL-RF combines Random Forest (RF) classifier and Positive and Unlabelled Learning (PUL) technique. RF was selected considering its high accuracy, efficiency and robustness in large-scale mapping by remote sensing^24^. PUL was employed because it can transform any binary classifier into a one-class classifier and avoid the need of labelled negative samples^46,47^. Such one-class classifier thus is more practicable than binary classifier when the negative class has a large diversity^48^, which is the case of non-PV class.

Firstly, classification features are derived from original Sentinel-2 data. Various spectral indices including NDVI, NDBI, and the two PV indices (i.e. NSPI-1 and NSPI-2) were calculated to enhance the spectral feature for PV classification. Here, NSPI-1 and NSPI-2 are designed based on the 1610 nm reflection peak feature of PV^11^

1 $${NSPI}-1=\frac{B11-B12}{B11+B12}$$

2 $${NSPI}-2=\frac{B11-B9}{B11+B9}$$

where B11, B12, and B9 are the reflectance values of B11, B12, and B9 in the Sentinel-2 band, respectively. Then, a bi-monthly synthesis procedure is applied to remove the cloud contamination in the time series of the spectral reflectance and the spectral indices. Thus, a total of 6 × 16 spectral-temporal features are derived from Sentinel-2 time series for classification.

Secondly, we establish specific training sample set for each subzone. In each subzone, a large number (i.e. 60000) of unlabelled samples are randomly generated considering no labelling cost is required. Stratified random sampling with land cover type strata (GlobeLand30) is implemented here to guarantee the inclusion of all land cove types in the unlabelled samples. Adequate positive samples (PV polygons generated in the first-stage classification), however, often cannot be acquired in one subzone because PV cover occupy a very small proportion of land surface or even do not exist in some subzones. Thus, the positive samples were acquired in the whole main zone that includes the target subzone. Such strategy is reasonable considering the spectral diversity of PV cover is much less than that of non-PV cover. Thus, PV samples selected from main zone can represent those in the subzone. With the generated training sample sets, specific PUL-RF classifier is trained for each subzone used for classifying the PV pixels from Sentinel-2 features. In order to reduce the randomness in training the RF classifier, we train the PUL-RF 10 times for each subzone and average the 10 classification probability outputs for the final classification. With the trained subzone-wise classifier, the global PV solar panel dataset with 20-meter resolution is obtained year by year from 2019 to 2022.

### Post-processing

The second-stage classification was implemented at the pixel level, so it inevitably suffers from “salt and pepper” errors. We use the inflationary erosion convolution for classification post-processing. As some asphalt roads and concrete roads have similar spectral features with PV, such post-processing effectively removed the misclassified line-like.

### Accuracy evaluation

In the first stage of U-Net-based PV sample generation, the accuracy evaluation metrics used are training accuracy and validation accuracy, which are used to evaluate the change in accuracy during the training process. Intersection over union (IoU) and Average precision (AP) were also used to evaluate the error between the U-Net extracted PV and the real PV.

In the second stage of PUL-RF-based PV extraction, the classification accuracy metrics including Overall Accuracy (OA), F1-Score, Producer Accuracy (PA), and User Accuracy (UA) are used for evaluating both classification and change detection accuracy based on the unchanged PV samples and new added PV samples respectively. We conducted accuracy evaluations on both the PVs existing in 2019 and the PVs newly added from 2019 to 2022.

## Data Records

The produced annual global PV dataset^49^ is publicly hosted on Zenodo (10.5281/zenodo.10684793) and GEE platform (https://cxh1216.users.earthengine.app/view/solarpv-bnu). It includes the annual global dataset of PV solar panels at 20-meter resolution from 2019 to 2022 in TIFF format. The validation data^38^ is publicly hosted on Zenodo (10.5281/zenodo.14348427), and the file type is vector file. The high resolution annotated PV sample set and the original GE images^33^ can be accessed in 10.5281/zenodo.14922538.

The folder of the annual global PV dataset is named after the year, and each file is named as “sub zoning ID_year”. The validation data file is named “validate_polygons. shp”. The naming of the original GE images is a continuous numbering system.

## Technical Validation

### Validation of annual PV classification maps

**Fig. 5:**
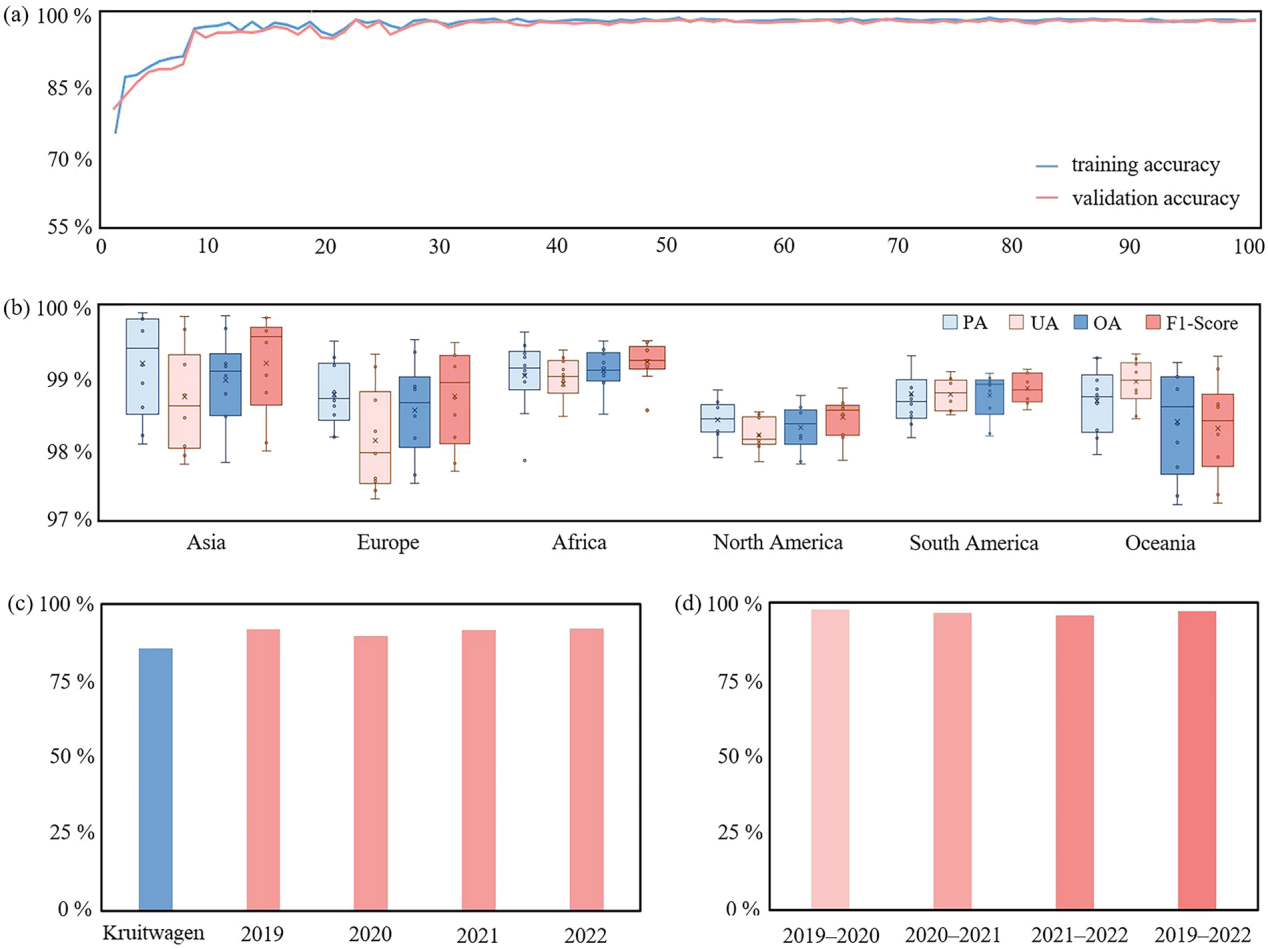
Accuracy evaluation results: (**a**) U-Net’s training accuracy and validation accuracy, (**b**) PUL-RF’s OA, PA, UA, and F1-Score, (**c**) the IoU in 2019–2022 compared to Kruitwagen’s IoU results, (**d**) the prediction accuracy (IoU) of newly added PV from 2019 to 2022.

In the first stage of U-Net-based PV sample generation, the segmentation accuracy of PV solar panels and background features are evaluated using IoU and AP, and the overall segmentation accuracy are evaluated using mIoU and mAP. The evaluation results are shown in Table 3, and the accuracy results all reach more than 98%. After training 100 epochs, the training accuracy and validation accuracy of U-Net can reach about 98% (Fig. 5a).

**Table 3:** Accuracy evaluation results of extracting PV based on U-Net in the first stage.

Classification type	IoU/mIoU	AP/mAP
Background	99.66%	98.91%
PV	98.15%	98.11%
Overall	98.89%	98.66%

We calculated the classification accuracy of PUL-RF in each continent partition (Fig. 5b). By continent, the model performs best in Asia, which has the highest classifier accuracy. Overall, the OA, PA, UA, and F1-Score all reach above 97%.

Comparing the accuracy of the new PV dataset from 2019 to 2022 with Kruitwagen’s dataset, the accuracy of the new dataset is over 90%, which is slightly higher than the dataset provided by Kruitwagen (Fig. 5c). The PV extraction method proposed in this study has high accuracy and stability on the validation set from 2019 to 2022, which implies that the two-stage classification framework can extract PV efficiently, and likewise indicates that the production of the new global PV dataset from 2019 to 2022 is reliable.

The accuracy of the new PV solar panels is evaluated for each time interval of 2019–2020, 2020–2021, and 2021–2022, as well as for the period 2019–2022 (Fig. 5d), and the calculation of the IoU shows that each year the IoU of new PV reaches more than 90%. The proposed PV extraction method shows high accuracy on the new PV in 2019–2022, which allows us to monitor the changes in PV solar panels.

**Fig. 6:**
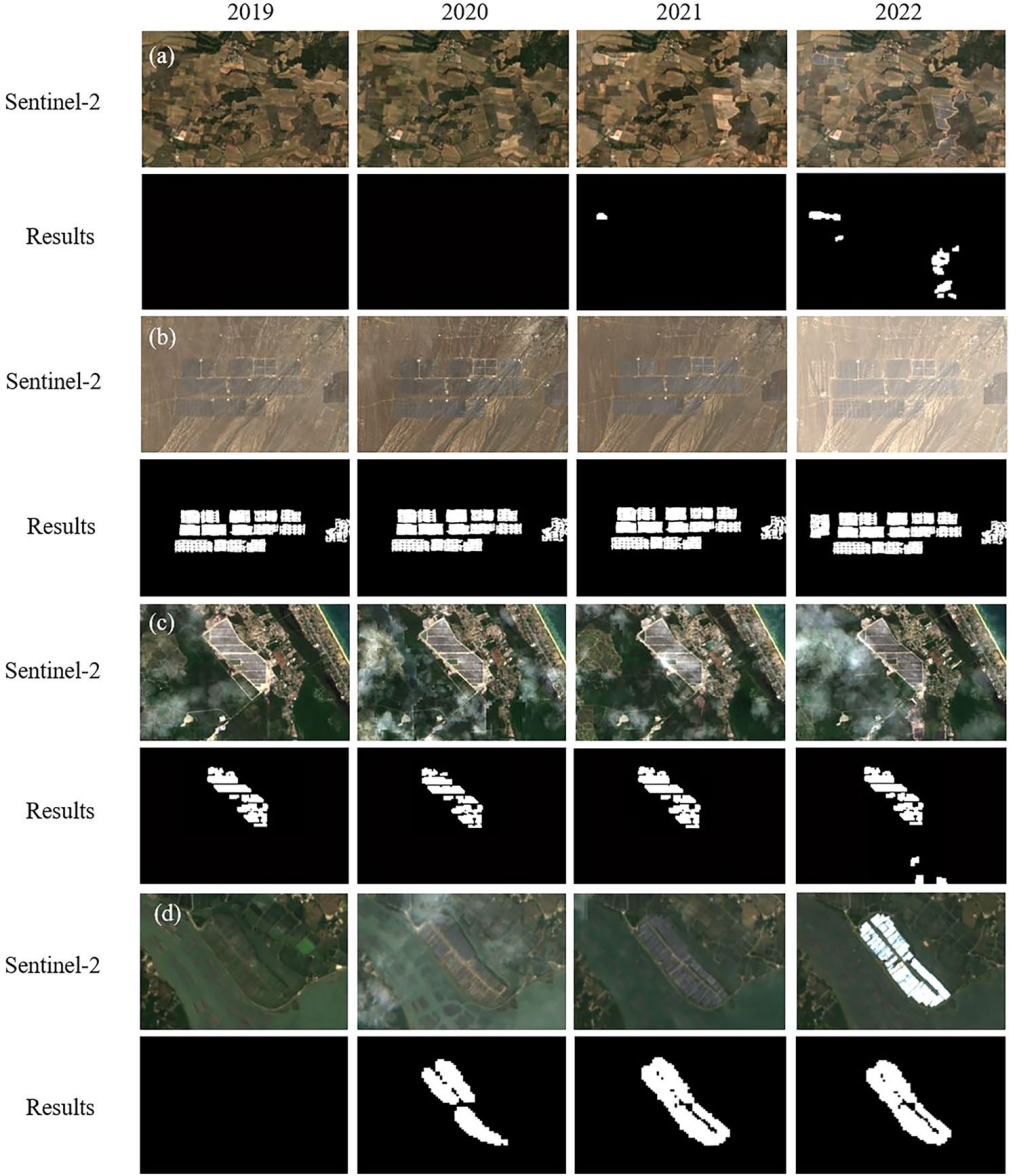
PV solar panel extraction results: (**a**–**d**) PV solar panels installed on cultivated land, bareland, forest and water bodies, respectively, Sentinel-2 images are 2019–2022 Sentinel-2 RGB images (median per bi-monthly composite). The locations of a-d are (2.640E, 43.218 N), (89.331E, 43.128 N), (103.336E, 4.935 N), and (108.476E, 21.846 N).

Due to the prior participation in training U-Net with PV solar panel labels covering various background types such as cultivated land, forest land, artificial surfaces, deserts, mountains, and water bodies, in the first stage, a relatively rich set of PV solar panels could be identified as positive samples for the second stage classification. In the second stage, when using PUL-RF, the classifier could learn the spectral characteristics of PV solar panels across multiple background types. Additionally, during the second stage of sampling, unlabelled samples were collected from various surface types, thus enabling the differentiation of solar panels from other surface types (Fig. 6). From the classification results, it can also be observed that when the background types are complex and diverse, the two-stage classification framework can effectively extract PV solar panels, particularly successfully distinguishing PV solar panels from high-brightness objects such as buildings. Moreover, the classification results of the PV solar panels align closely with the actual characteristics of solar panel installations and PV arrays, accurately delineating the clear contours of solar panels and the gaps between contiguous solar panels.

The 20-meter resolution global PV solar panel mapping dataset we produced for 2019–2022 captures the changes in PV during this period. From the changes in the Sentinel-2 imagery in Fig. 6(a), it can be observed that solar panels began to be installed on cropland in this area in 2021, and by 2022, the area of solar panels had expanded. The classification results in Fig. 6(a) accurately capture this feature. From the Sentinel-2 imagery in Fig. 6(b,c), it is evident that these two places had solar panels as early as 2019, and there was an expansion in the scale of the solar panels from 2020 to 2022. Figure 6(d) also shows the changes. The classification results not only extract the scale of PV solar panels in 2019 but also present the annual changes in the solar panels.

In the temporal analysis of global PV solar panels for 2019–2022, the global area of PV solar panels for each year 2019–2022 was first counted. In 2019 the global area of PV was 3831.6 km^2^, and in 2022 the area of PV grows to 6469.8 km^2^, the growth is 2638.2 km^2^. The overall growth rate of PV solar panel area is more than 60%. We counted the PV solar panel area in the world and the ten countries with the largest PV solar panel area in 2019–2022 (Table 4), and China is the country with the largest amount of PV and the largest amount of PV growth, with a PV area of 2542.5 km^2^ and a growth of 1014.6 km^2^ by 2022.

**Table 4:** PV area of the top ten countries with the highest PV area globally from 2019 to 2022.

Country	2019 (km^2^)	2020 (km^2^)	2021 (km^2^)	2022 (km^2^)
Global	3831.6	4334.4	5030.3	6469.8
China	1527.9	1674.8	1993.5	2542.5
US	573.8	664.9	745.2	1051.4
India	407.5	435.3	533.7	673.9
Germany	171.6	181.1	195.2	248.1
Japan	139.4	140.8	172.2	214.9
Mexico	67.8	78.1	82.2	132.5
Australia	64.3	105.7	115.0	128.3
Spain	52.7	65.8	88.6	117.6
UK	90.8	92.3	94.4	105.5
Chile	44.4	53.5	62.6	100.3

**Fig. 7:**
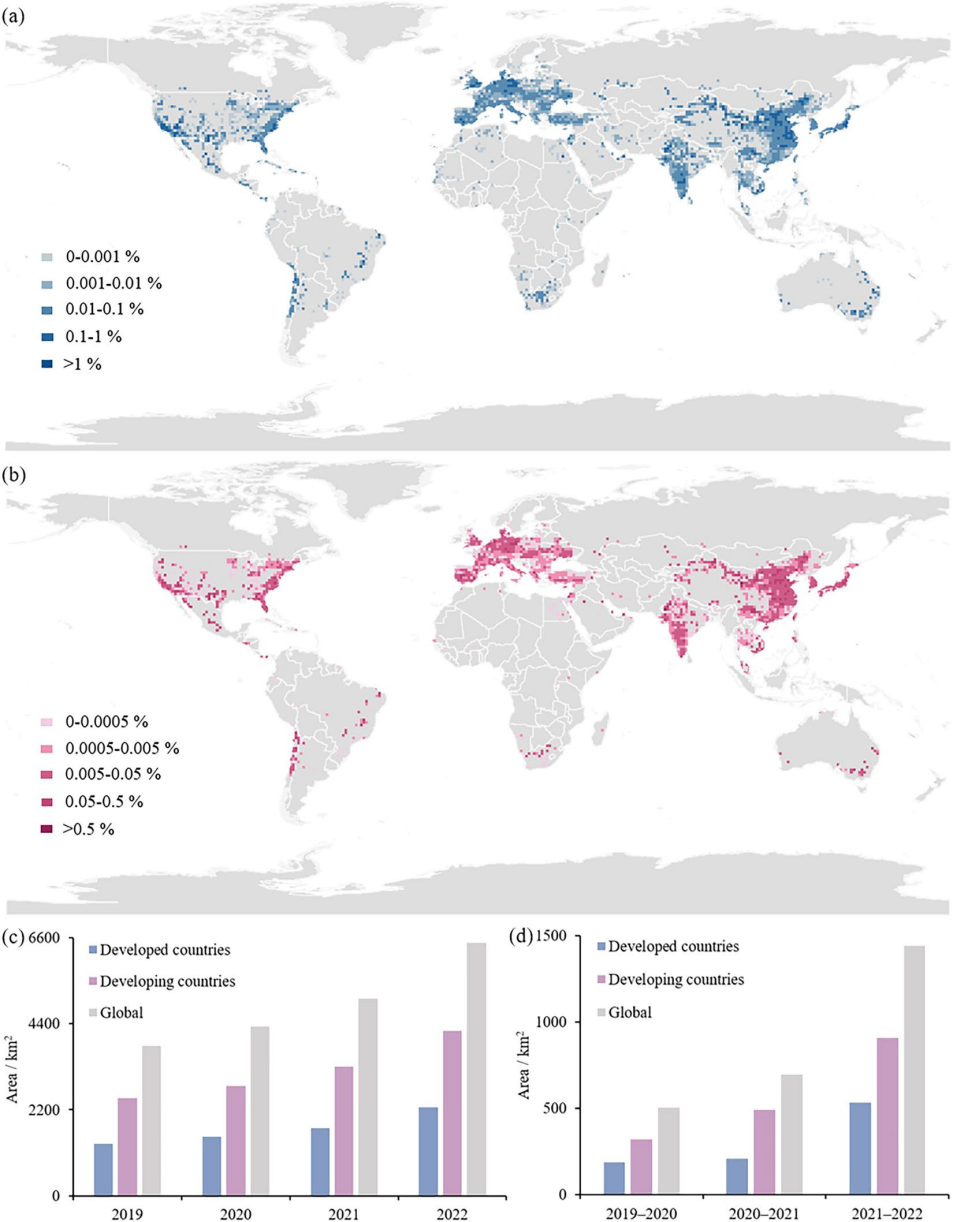
Global PV distribution and change: (**a**) the global PV distribution in 2022, (**b**) the distribution of new PV from 2019 to 2022, (**c**) the PV area of developed and developing countries from 2019 to 2022, (**d**) newly increased PV area of developed and developing countries from 2019 to 2022 (the map is made with a 1-degree grid to show the global PV distribution, showing the proportion of PV area in each grid).

In presenting the global PV distribution and changes, in order to visualize the 20-meter resolution global PV dataset we produced for 2019–2022, we counted and mapped the global PV area and growth status using 1-degree grids as the statistical unit (Fig. 7). Considering that the deformation generated by the map projection will lead to the difference in the area of each 1-degree grid, we do not use the area to quantify each 1-degree grid, but rather count the proportion of PV contained in each 1-degree grid in this grid, so as to statistically compare the PV that exists in each 1-degree grid, which is a more rigorous way of mapping.

From the spatial distribution characteristics of PV solar panels in 2022 (Fig. 7a), global PV is concentrated in the middle and low latitudes, and there is little PV distribution in high latitudes. Besides, PV solar panels are mostly distributed in densely populated areas of the world except Africa. In terms of each continent, Asia, Europe, and North America have a relatively large number of PV installations, whereas South America, Africa, and Oceania have a relatively small number of PV installations. PV in South America and Oceania is mainly concentrated along the South Pacific coast. In terms of the density of PV deployment, Europe and Asia have the highest PV density and the most concentrated distribution, with the Mediterranean coast of Europe and East Asia having the highest PV density, with PV solar panels distributed in almost every 1-degree grid. In addition, the distribution of PV in the eastern and western coasts of North America is also relatively concentrated. In contrast, Africa’s PV distribution is more decentralized, and the PV area is smaller, mainly concentrated in southern Africa, north of the Sahara Desert, which also has a small amount of PV distribution.

Global PV solar panel growth is evident during 2019–2022. In terms of the spatial distribution characteristics of PV solar panel growth (Fig. 7b), mid- and low-latitude regions have more PV growth. In terms of hotspots for PV additions, the Mediterranean coast of Europe, Central Europe (Germany), East Asia (China), South Asia (India), Southeast Asia, and the east and west coasts of North America have more pronounced hotspots for PV additions. Among them, Asia and Europe have a large number of new PV, and the density of new PV in Europe and Asia is also relatively high. In contrast, new PV in Africa is very dispersed, with almost no new centralized PV solar panels installed in Africa during 2019–2022, except in South Africa and Egypt.

Statistics on the area and change of PV solar panels in developed and developing countries from 2019–2022 show that the PV area in developing countries is much larger than that in developed countries (Fig. 7c,d). As of 2022, developing countries own 4211.2 km^2^ of PV, which is about twice as much as developed countries, and the amount of new PV in developing countries is also higher than that in developed countries, with the new PV area in developing countries being 1713.3 km^2^. From this observation, PV in developing countries is ahead of that in developed countries, and it is the mainstay of the world’s PV market.

### Comparison with existing global PV datasets

**Fig. 8:**
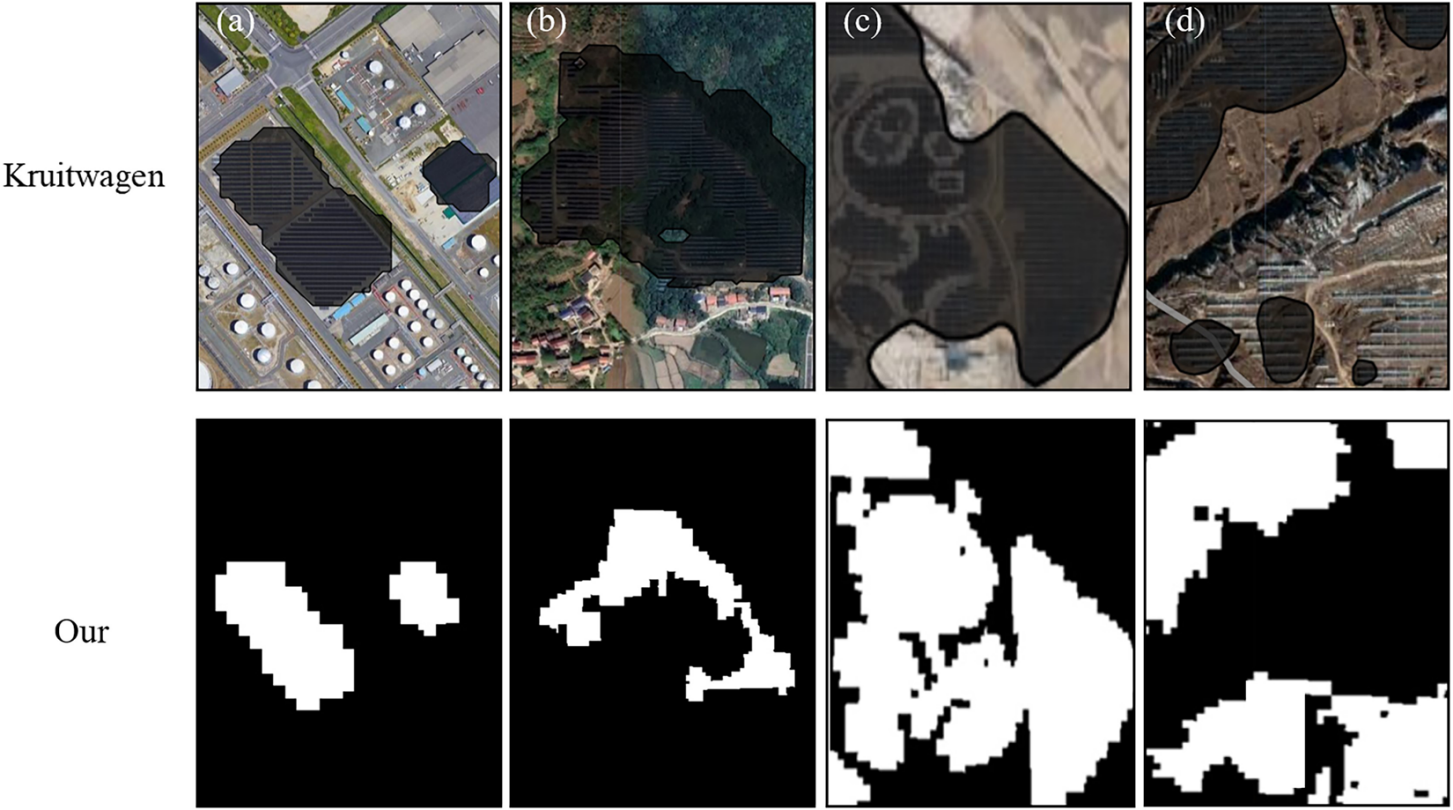
Comparison of the Kruitwagen’s dataset (shaded) and the new dataset: (**a**–**d**) solar panels installed on artificial surfaces, forest, grassland, and bareland, respectively (the high-resolution images are from © Google Earth 2022).

Comparing the produced 2019–2022 PV dataset with the currently sole global solar panel spatial dataset^30^ (Kruitwagen *et al*., 2021), the new dataset achieves an IoU of over 90% for PV in each year, surpassing the IoU of Kruitwagen’s dataset (Fig. 5c). In a partial comparison between the new dataset and Kruitwagen’s dataset (Fig. 8), although Kruitwagen’s results can extract the basic information of PV solar panels, the extracted range is larger than the actual range of PV solar panels and cannot distinguish the gaps between PV arrays. Furthermore, the new dataset performs better when the PV arrays are sparse. In contrast, the range of PV solar panels identified by the new dataset is more accurate.

Kruitwagen’s vector format dataset emphasizes patch continuity through polygon boundaries, whereas our product employs a pixel-level raster format. This comparison should not be interpreted as an accuracy assessment, but rather seeks to highlight the necessity for (1) recognizing these representational discrepancies, and (2) implementing appropriate data harmonization techniques when performing temporal analysis. Researchers working with the two products should particularly consider these format-specific characteristics during analytical workflows.

## Usage Notes

While we are able to perform a complete extraction for centralized PV solar panels, and the extraction accuracy is higher than the currently available global PV solar panel dataset, there are some errors in the identification results for smaller distributed and rooftop PV. The source of the error is the limitation of Sentinel-2 imagery. Smaller scale distributed and rooftop PV solar panels are often made up of several solar panels spliced together with a small area, not even up to the size of a single image element on a Sentinel-2 image. In the future, it will be necessary to consider the use of higher-resolution remote sensing imagery or estimation methods to extract these smaller-scale solar panels. For example, using higher resolution images to extract roof PV. Although there are errors in the extraction results of this dataset for some of the smaller distributed PVs, some of the larger distributed PVs were able to be extracted in a more complete manner, and for a portion of the rooftop-mounted solar panels that can be grid-connected are able to be represented in the dataset.

## Data Availability

The fully reproducible codes^50^ are available at 10.5281/zenodo.15168340.
